# Graphene Oxide-Modulated Nanocellulose/Polyacrylamide/Sodium Alginate Hierarchical Network Hydrogel for Flexible Sensing

**DOI:** 10.3390/gels11060379

**Published:** 2025-05-22

**Authors:** Yanan Wang, Yanan Lu, Jiaming Wang, Chensen Huang, Minghui Guo, Xing Gao

**Affiliations:** 1 Key Laboratory of Bio-Based Material Science and Technology of Ministry of Education, Material Science and Engineering College, Northeast Forestry University, Harbin 150040, China; ynwangnefu@163.com (Y.W.); 15948889537@163.com (J.W.); hcs2287130165@163.com (C.H.); 2 Institute of Vegetables and Flowers, Inner Mongolia Academy of Agricultural and Animal Husbandry Sciences, Hohhot 010000, China; ynlunefu@163.com; 3 School of Kinesiology and Health, Harbin Sport University, Harbin 150008, China

**Keywords:** hydrogel, nanocellulose, hierarchical network, flexible sensing, graphene oxide

## Abstract

The application of hydrogels in flexible sensing has received increasing attention, but the simultaneous preparation of hydrogels with good structural stability, strain sensing sensitivity, freezing resistance, and drying resistance remains a challenge. Based on this, a GG-nanocellulose/sodium alginate/polyacrylamide composite hydrogel with a hierarchical network structure was constructed by one-step synthesis by incorporating graphene oxide (GO) and glycerol into the hydrogel. The hydrogel remained structurally intact after 100 compression cycles. In addition, the hydrogel was dried at 30 °C for 24 h. The mass retention rate was 48%, the melting peak was as low as −13.87 °C, and the hydrogel remained flexible and stable at low temperatures. GO modulated the network structure arrangement of the hydrogel through various mechanisms, thereby conferring to the hydrogel an excellent sensing performance, with a sensitivity (GF) of 2.21. In conclusion, this hierarchical network hydrogel has good drying, freezing, and sensing properties, which provides a new viable strategy for monitoring motion signals. Moreover, the hydrogel is predicted to function as a dressing, thereby facilitating the absorption of heat from the skin’s surface, with the aim of alleviating the discomfort associated with joint and muscle injuries caused by strenuous exercise.

## 1. Introduction

Research on flexible stretchable sensors is currently moving towards high performance, multifunctionality, and intelligence, with their compliance and ability to shape at will allowing them to respond to changes in the human body and to perform tasks that are not possible with rigid sensors [1]. Among them, hydrogels have become one of the key research directions in flexible sensing due to their good flexibility, self-healing, and adhesion properties [2]. Furthermore, hydrogels are amenable to functional customisation, with the capacity to exhibit properties such as frost and heat resistance, rendering them suitable for utilisation in extreme climatic environments [3,4]. In addition to areas such as wearable monitoring sensing, many researchers and scholars have worked to improve the sensing performance while making it functional for motion protection [5,6]. Hydrogel as a flexible sensor has its unique advantages in terms of its biocompatibility, flexibility, and sensitivity [7,8], Nevertheless, its poor resistance to drying, single-stimulus-response characteristics, and inability to be utilised in extreme environments have severely limited the application of hydrogel sensors. These problems can be improved by introducing materials such as conductive polymers [9], nanoparticles [10], low-eutectic solvents [11,12], liquid metals [13], and ionic liquids [14,15] to achieve high sensitivity, a wide detection range, and excellent fatigue resistance.

Nanofibrillar cellulose (CNF) has been gradually introduced into gels for flexible sensing applications in recent years due to its natural renewability, good biocompatibility, and abundant sources [16,17]. CNF has a high specific surface area, excellent mechanical strength, and degradability, and the hydrogel formed by combining it with many functional components combines high elasticity, electrical conductivity, and tuneable sensing properties, mainly focusing on signal monitoring, using its high sensitivity and biocompatibility, real-time muscle movement, joint movement, and sweat metabolites, which are suitable for wearable health monitoring devices [18,19,20,21]. CNFs are generally interspersed in the polymer hydrogel network by interpenetration [22,23] or semi-interpenetration [24,25,26], and the linear network structure formed can effectively improve the tensile toughness and ductility properties of the hydrogel after cross-linking with the polymer network. In the study of Zhi Yang et al., the compressive stress of the sensing gel increased from 0.71 MPa to 0.92 MPa after the addition of CNF [27], and Cancan Shan et al. increased the Young’s modulus of the gel from 30.51 kPa to 140.505 kPa by increasing the amount of CNF added in the preparation of the sensing gel [28]. The preceding studies demonstrate that CNF can effectively improve the mechanical properties of gels, and good mechanical properties are one of the important indexes for judging the performance of sensing gels.

In addition, the addition of carbon materials is also an important way to improve flexible sensing hydrogels [29,30,31,32]. In recent years, graphene oxide (GO) has a wide range of applications in the fields of electronics, energy storage, and sensing by virtue of its high specific surface area and abundant oxygen-containing functional groups [33]. As a two-dimensional nanomaterial can be guided by its lamellar structure to an ordered arrangement of gel molecules [34] to form an ordered porous network, it can optimise the network structure and anisotropy of gels and significantly enhance their performance in terms of structural stability, electrical conductivity, and sensing [35].

Based on this, in this study, a dual network GG-CNF/SA/PAM hydrogel with enhanced stability was constructed for flexible sensing application by using GO to modulate the directional arrangement of the hydrogel network structure, which also possesses good moisture retention and anti-freezing properties. CNF is uniformly distributed throughout the cross-linked network in a linear manner, and graphene oxide improves the mass transfer capacity and sensing performance of the hydrogel by adjusting the hydrogel network arrangement. The prepared GG-CNF/SA/PAM can be used as a flexible sensor to feedback signals according to different movements. In addition, the hydrogel can be used as a dressing to effectively absorb heat to relieve joint and muscle injuries caused by exercise.

## 2. Results and Discussion

### 2.1. Micro-Morphological Analysis of Hydrogels

**Figure 1:**
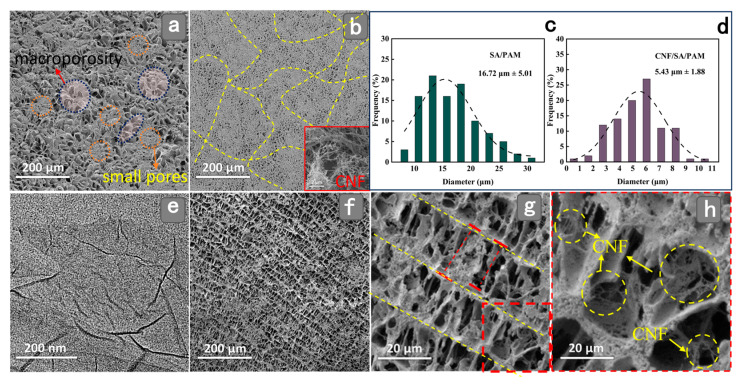
SEM image of SA/PAM (**a**); SEM image of CNF/SA/PAM (**b**); average pore size of SA/CNF (**c**); average pore size of CNF/SA/PAM (**d**); TEM image of GO (**e**); cryo-EM image of GG-CNF/SA/PAM (**f**); magnified structure of GG-CNF/SA/PAM (**g**); pore microscopic magnification of GG-CNF/SA/PAM (**h**).

The microscopic morphology of the hydrogel is shown in Figure 1. The cross-linking of polyacrylamide (PAM) and sodium alginate (SA) was used as the basic hydrogel system, and the composite of the two polymers could effectively improve the cross-linking stability of the hydrogel and form a uniform staggered double network structure (Figure 1a). As shown in Figure 1b, when CNF was introduced, the pore size of the hydrogel was reduced, the pore layout was more uniform, and the bonding between the interfaces was tighter. This is due to the spatial site resistance effect of CNF [36], which modulates the overall network structure of SA/PAM. In addition, since CNF mainly exists as spiderweb-like nanofibrils, it can be used as a linear structure and a double network structure interspersed with each other to form a semi-interpenetrating network structure, which is conducive to the improvement in the stability of the network structure of the hydrogel. Meanwhile, as shown in Figure 1b,c, the pore size was also found to be reduced from 16.72 μm to 5.43 μm by calculation, which again indicates that the incorporation of CNF greatly shrinks the network structure of the hydrogel. On this basis, the surface of the introduced propanetriol carries a large number of hydroxyl groups, which can be combined with the functional groups on the surfaces of SA, CNF, and PAM with good hydrogen bonding, and improve the hydrogen bonding cross-linking ability between different components of the hydrogel. Graphene oxide (GO) (Figure 1e) can be observed by transmission electron microscopy in a nanoscale lamellar structure. GO is a two-dimensional material composed of a single layer of carbon atoms, which can first act as a stencil or scaffold, acting in synergy with CNF through spatial site resistance and electrostatic interactions, leading to an ordered arrangement of the gel network structure (Figure 1f) [37]. Furthermore, the unique network structure may also be attributed to the strong interaction inhibition of the mobility of the polymer chains when GO is combined with PAM, CNF, and so on (Figure 1g,h) [38].

### 2.2. Chemical Structure Analysis of Hydrogels

**Figure 2:**
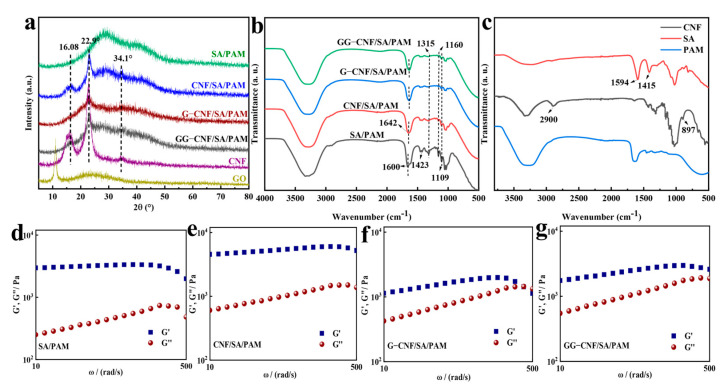
XRD pattern (**a**); Fourier transform infrared (FTIR) spectrum (**b**,**c**); rheological properties of SA/PAM (**d**), CNF/SA/PAM (**e**), G-CNF/SA/PAM (**f**), and GG-CNF/SA/PAM (**g**), where G′ is the storage modulus and G″ is the loss modulus.

The XRD patterns of the hydrogels were tested to observe the binding of the hydrogels as shown in Figure 2. As shown in Figure 2a, 2θ = 28.68° is the broad diffraction peak of SA/PAM. When CNF was added to the hydrogel, the typical characteristic peaks of cellulose appeared at 2θ = 16.39, 22.9°, and 34.1°, while the broad diffraction peaks of SA/PAM were retained [39]. The intensity of the diffraction peaks of cellulose decreased after the addition of glycerol and GO, which was attributed to the multiple interactions between GO and the polymer chains [40]. Due to the relatively small amount of GO added, no obvious diffraction peaks of GO were found in the XRD patterns. In addition to this, the interactions between the components in the hydrogel were characterised using FTIR. As shown in Figure 2b,c, when PAM and SA were combined, the antisymmetric telescopic vibrational absorption peak of -COO- at 1594 cm^−1^ in SA disappeared, the symmetric telescopic vibrational absorption peak of -COO- at 1415 cm^−1^ in SA was shifted to 1423 cm^−1^, and, at the same time, the intensity of the diffraction peaks was significantly reduced, which may be attributed to the effect of the hydrogen bonding between PAM and SA [30,41]. After the addition of CNF, the absorption peak of SA/PAM at 1660 cm^−1^ is a stretching vibration of C=O in PAM (amide I band) moving to 1642 cm^−1^, which may be due to hydrogen bonding between PAM, SA, and CNF [42]. After the addition of glycerol and GO, the peak of SA/PAM at 1660 cm^−1^ gradually shifted to the right, and the positions of other diffraction peaks were also slightly shifted. Those at 1315 cm^−1^ (amide III band) indicate C-N stretching vibration absorption peaks, and the intensities of the C-O stretching vibration absorption peaks of SA at 1160 and 1109 cm^−1^ were weakened but still retained the functional groups of each component. This is due to the non-covalent interactions between the abundant functional groups carried by multiple components [30].

The rheological properties of the hydrogels were tested using a rotational rheometer. Since CNF is a nanoscale fibrous material with high aspect ratio and high mechanical strength, the storage modulus (G′) was significantly higher than the loss modulus (G″) when CNF was added to the SA/PAM network, as shown in Figure 2d,e. This is due to the spatial barrier effect of CNF, which makes the fibre filaments uniformly dispersed in the network structure, and hydrogen bonding or physical entanglement with SA and PAM improves the mechanical strength of the hydrogel [43]. After the addition of glycerol to the CNF/SA/PAM hydrogel network, as shown in Figure 2f, G′ and G″ begin to weaken and intersect in the high-frequency region, and the rigidity strength of the hydrogel is significantly reduced. This is due to the plasticising effect of glycerol exhibiting the ability to reduce the self-entanglement of CNF, enhance the fluidity of the hydrogel, and endow the hydrogel with stronger flexibility and tensile properties [28]. When GO was added to G-CNF/SA/PAM, as shown in Figure 2g, the plasticising effect brought by glycerol was appropriately alleviated, so the hydrogel had good tensile properties while maintaining flexibility.

### 2.3. Reaction Mechanism Diagram of GG-CNF/SA/PAM

**Figure 3:**
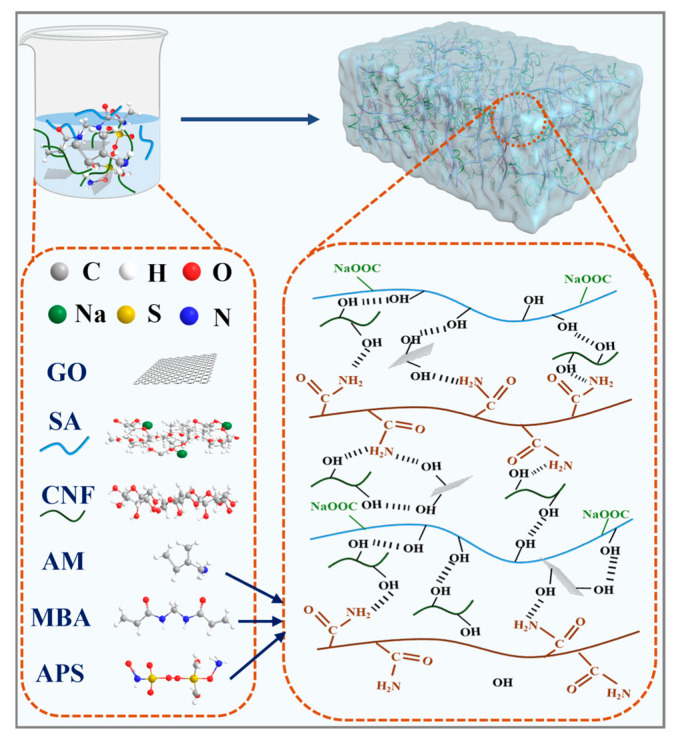
Reaction mechanism diagram of GG-CNF/SA/PAM.

Figure 3 shows the synthesis mechanism of hydrogels. GG-CNF/SA/PAM semi-interpenetrating network hydrogels were constructed by the one-step synthesis of ammonium persulfate (APS) as an initiator and *N*,*N*′-methylenebisacrylamide (MBA) as a cross-linker. Firstly, it is important to note that the long fibres of CNF have the capacity to intertwine with SA/PAM chains, thereby forming physical cross-linking points. In addition, the hydroxyl group of CNF is capable of forming multiple hydrogen bonds with SA, PAM, and GO, thus enhancing the mechanical strength of the hydrogel [28]. Secondly, the amide group (-CONH_2_) of PAM interacts with the polar groups of SA, CNF, and GO, as evidenced by the characteristic peak shift near 1600 cm^−1^ in the FTIR pattern. Following GO incorporation, the oxygen-containing functional groups of the GO can also form hydrogen bonds with SA, PAM, and CNF. Furthermore, CNF and GO can modulate the homogeneity of the synthesised gels through spatial site resistance and electrostatic interaction [44]. Since the dual mechanism of CNF and GO provides an effective energy dissipation mechanism for the network structure, when the hydrogel is subjected to an external force, the CNF, GO, and polymer chains undergo sliding and rearrangement accompanied by a rapid reorganisation of the network, which results in a flexible, tensile, ductile, and malleable gel [45,46,47].

### 2.4. Analysis of Mechanical Properties of Hydrogels

**Figure 4:**
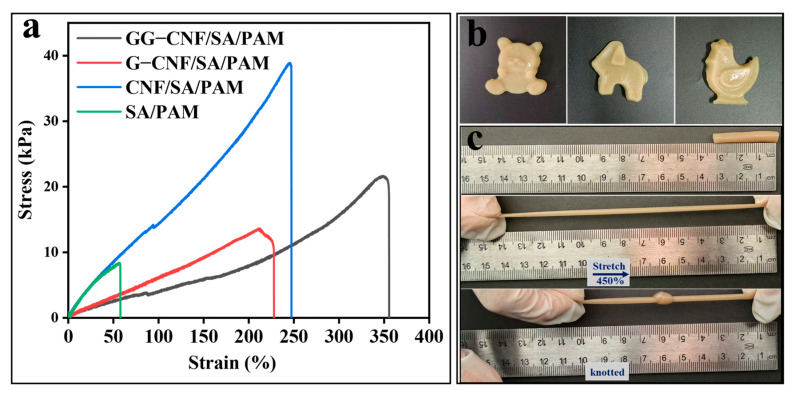
Stress–strain curves of hydrogels in the tensile state (**a**); GG-CNF/SA/PAM prepared in different moulds (**b**); a physical diagram of GG-CNF/SA/PAM in the tensile state (**c**).

The tensile properties of the hydrogels are shown in Figure 4a. The introduction of CNF effectively improved the tensile strength of SA/PAM, the tensile stress of the hydrogels was increased from 8.31 MPa to 38.91 MPa, and the tensile strain was changed from 58% to 248%. The introduced CNF was uniformly distributed inside the hydrogel in a linear manner, which provided a certain degree of support for the hydrogel and acted as a scaffold to resist the external compressive stress. In addition, CNF improved the mechanical properties of the gels with hydrogen bonding between the polymers [27]. The addition of glycerol and GO can make the hydrogel network shift to an orderly directional arrangement, and the directional arrangement of the network structure can offset some of the stress in different directions. The main reason for this hierarchical network structure to counteract the stresses is due to the high-aspect-ratio fibres of the CNF absorbing energy by stretching or slipping [48]. Furthermore, GO also undergoes lamellar slip or rearrangement under stress to disperse multidirectional stress [49]. The incorporation of glycerol serves to minimise the rigid contact points of SA/PAM chain segments, thereby mitigating the potential for crack formation due to stress concentration. As shown in Figure 4b,c, GG-CNF/SA/PAM has excellent plasticity, and different hydrogel morphologies can be prepared according to different application requirements. The above results indicate that the prepared GG-CNF/SA/PAM hydrogels have excellent tensile stress–strain properties.

**Figure 5:**
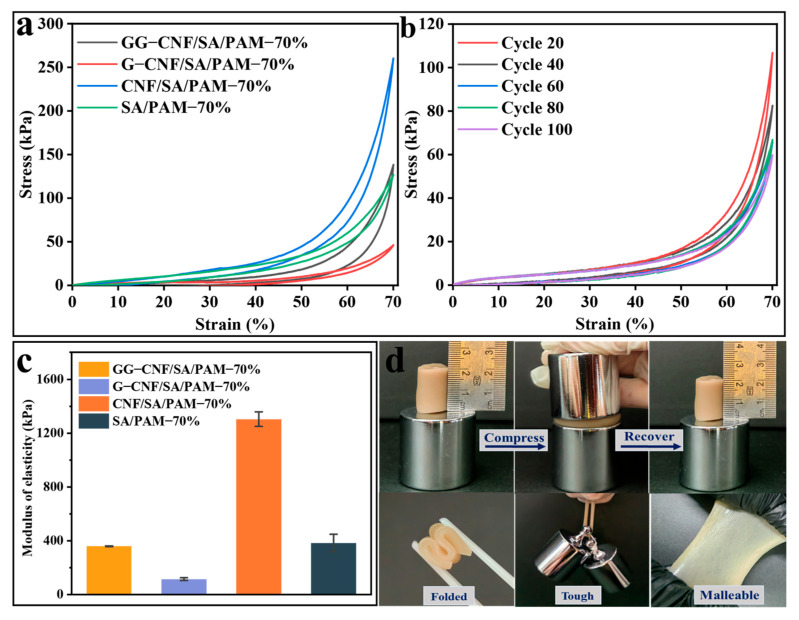
Resilience properties of hydrogel compressed by 70% (**a**); GG-CNF/SA/PAM at 100 compression cycles (**b**); modulus of elasticity of hydrogel in 70% compression (**c**). Physical diagram of GG-CNF/SA/PAM in different states (**d**).

The compressive properties of the hydrogels are shown in Figure 5a. All hydrogel samples possessed compression-resilience properties at 70% strain. Among them, the compressive stress of CNF/SA/PAM at 70% compression is 5.8 times higher than that of SA/PAM. In order to test the structural stability of GG-CNF/SA/PAM during repeated compression, GG-CNF/SA/PAM was compressed–rebounded 100 times at 70% of compression deformation. As shown in Figure 5b, when the compression cycle is 20 times, the compression stress is 106 kPa; when the cycle is 40 times, the compression stress is 81 kPa; when the compression is 60 and 80 times, the compression stress is around 66 kPa; and when the compression cycle reaches 100 times, the compression stress can still be maintained at 60 kPa, which shows that the GG-CNF/SA/PAM has a good structural stability. The modulus of elasticity (Figure 5c) also demonstrates that the addition of CNF effectively increased the rigidity of the hydrogel, while the addition of GO and glycerol modulated the elasticity of the hydrogel. Therefore, as shown in Figure 5d, the GG-CNF/SA/PAM hydrogel can be restored to its original shape even after a huge deformation under compression, and the external shape of the hydrogel is not affected in any way. In addition, GG-CNF/SA/PAM can be easily folded and lifted with weights. The above results show that the hierarchical network structure of GG-CNF/SA/PAM can make the hydrogel undergo different deformation states during stretching and compression, and this structure can make the hydrogel have more possibilities in the process of application.

### 2.5. Analysis of the Anti-Drying Properties of Hydrogels

**Figure 6:**
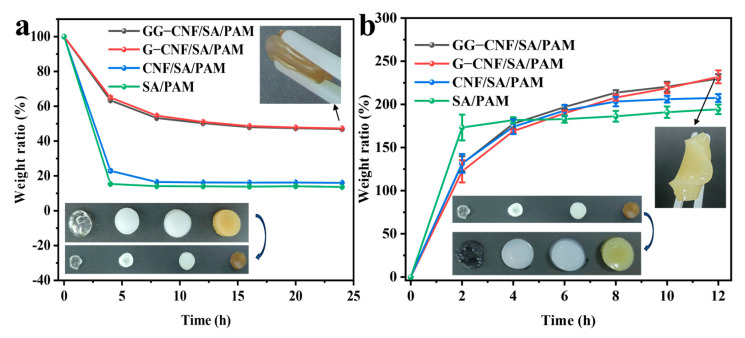
Weight loss rate of hydrogel (**a**); swelling rate of hydrogel (**b**).

The anti-drying property is one of the important advantages of hydrogels, which is of great significance for sensing performance transmission, environmental adaptability, and application stability. The water loss characteristics of different hydrogels are shown in Figure 6a, which shows that the weight of SA/PAM decreased by 86.4%, the weight of CNF/SA/PAM decreased by 83.9%, and the weights of G-CNF/SA/PAM and GG-CNF/SA/PAM only decreased by about 52% after 24 h in the oven at 30 °C, suggesting that the water retention property of hydrogels is mainly improved by the incorporation of glycerol in the hydrogels [50]. The incorporation of glycerol can inhibit the evaporation of water from the hydrogel due to the strong hydrogen bonding interactions between glycerol and water, as well as the lower volatility and higher boiling point of glycerol [51]. Concurrently, the interfacial enhancement due to the incorporation of CNF and GO also helps to lock in the water, while the gels with the addition of glycerol also absorb more water from the environment for the maintenance of the moisture content of the hydrogel [52]. The physical picture also shows that GG-CNF/SA/PAM maintains good softness after 24 h of drying. In order to test the stability of the hydrogel network, the hydrogel that lost water for 24 h was soaked in deionised water to observe the recovery of the hydrogel network. From Figure 6b, it can be seen that the GG-CNF/SA/PAM hydrogel has the best swelling property compared to other samples in the same condition. This is due to the large number of hydrophilic groups in the hydrogel components that generate osmotic pressure in water, driving water into the hydrogel and causing it to swell. This is an advantage of hydrogen bonding cross-linking to build the hydrogel network, which can give the hydrogel re-swelling properties.

### 2.6. Analysis of Anti-Freezing Properties of Hydrogels

**Figure 7:**
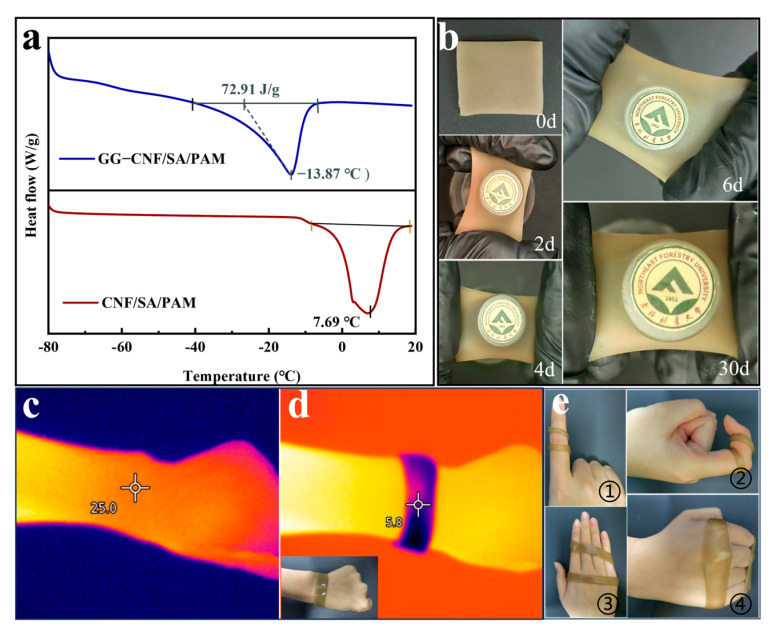
DSC curves of CNF/SA/PAM and GG-CNF/SA/PAM (**a**); the state of GG-CNF/SA/PAM frozen for different days (**b**); the temperature at the wrist at room temperature (**c**); the temperature at the wrist after adhering the hydrogel at the wrist for 2 min (**d**); GG-CNF/SA/PAM fits onto different positions of the hand (**e**) (① hydrogel twisted around a straightened finger, ② Hydrogel twisted around a bent finger, ③ hydrogel twists around the outstretched palm, ④ hydrogel twists around the curved palm).

Hydrogel’s anti-freezing performance is also one of the important application indexes, with the good anti-freezing performance of hydrogel allowing it to function in low-temperature, sub-zero, and other relatively extreme environments, thus exhibiting good application value. From Figure 7a, the melting peak moved from 7.69 °C for CNF/SA/PAM to −13.87 °C for GG-CNF/SA/PAM, indicating that GG-CNF/SA/PAM has good anti-freezing performance. This is due to the fact that glycerol can form strong hydrogen bonds with water molecules and inhibit the growth and aggregation of ice crystals [53,54]. To verify the longevity of the anti-freezing property of the hydrogel, as shown in Figure 7b, the state of the hydrogel was viewed after placing the hydrogel in a refrigerator (−20 °C) at 2, 4, 6, and 30 days, and the results showed that the hydrogel was still soft and elastic even after 30 days of freezing. This kind of hydrogel with long-lasting anti-freezing properties is beneficial for flexible sensing applications in extreme environments. Moreover, hydrogel is also expected to be used as a cold compress after soft joint strains. The comparison of Figure 7c,d indicates that the temperature of the wrist decreased from 25 °C to 5.8 °C after the hydrogel was cold-applied to the wrist. Compared with traditional cold application methods (ice packs, ice cubes, etc.), hydrogel can regulate the temperature by freezing, and the cooled hydrogel absorbs the heat from the skin surface to achieve the cold application effect. The fact that hydrogels are randomly shaped irrespective of temperature and can be reused is also one of the advantages of their use in sports protection and dressings (Figure 7e).

### 2.7. Analysis of the Sensing Properties of Hydrogels

**Figure 8:**
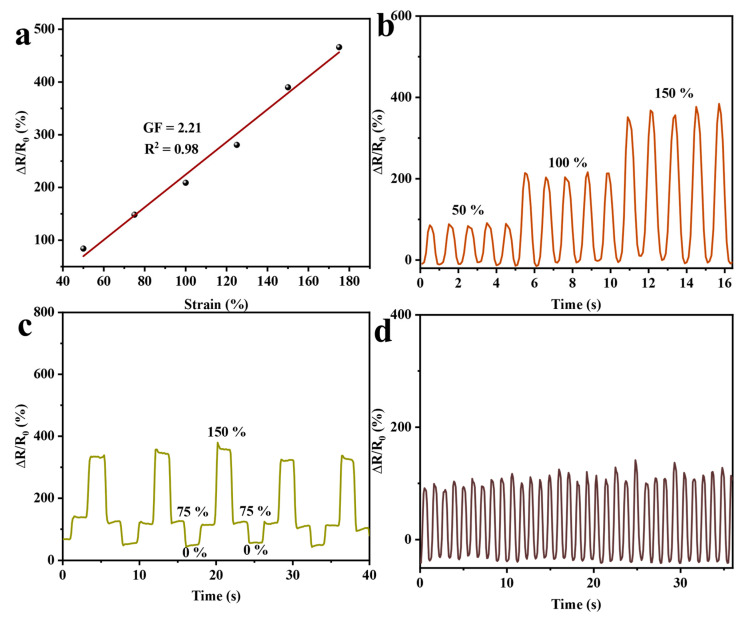
GF values of GG-CNF/SA/PAM with corresponding strain (**a**); GG-CNF/SA/PAM during stretch–recovery cycles at strain (50–150%) (**b**); relative resistance changes of GG-CNF/SA/PAM repeatedly stretched to 75% and 150% (**c**); GG-CNF/SA/PAM at strain of 50% for multiple stretch-cycling (**d**).

This kind of hydrogel with a hierarchical network structure with excellent deformation ability and anti-drying and anti-freezing properties has good prospects in the field of flexible sensing if it has good sensing properties. Figure 8 investigates the relative resistance change (∆R/R_0_) with tensile strain. As illustrated in Figure 8a, ∆R/R_0_ increases with the tensile strength of the strain, and the sensitivity of the hydrogel also increases. Based on the fitted curves, it can be seen that the sensitivity (GF) of GG-CNF/SA/PAM reaches 2.21. As in Figure 8b, the hydrogel was continuously stretched to 50%, 100%, and 150% of the corresponding lengths, and ∆R/R_0_ increased to 100%, 200%, and 375%, respectively. The signal transmission was stable over multiple stretch–release cycles. As demonstrated in Figure 8c, GG-CNF/SA/PAM can repeatedly reproduce the change in ∆R/R_0_ under strains of 75% and 150% in tension and maintain the relative resistance during the change. Figure 8d shows that GG-CNF/SA/PAM has long-term stability in resistance change under fixed deformation. This is attributed to the excellent electrical conductivity of GO, which is highly sensitive to external stimuli, and enhances the sensitivity of the hydrogel. Secondly, the hierarchical aligned network structure of the hydrogel maintains a high degree of ductility and elasticity under a variety of synergistic interactions, which facilitates the good fatigue resistance of the hydrogel in continuous tensile cycling, and ensures a good conductive pathway for GG-CNF/SA/PAM [55].

### 2.8. Application Analysis of Hydrogels

**Figure 9:**
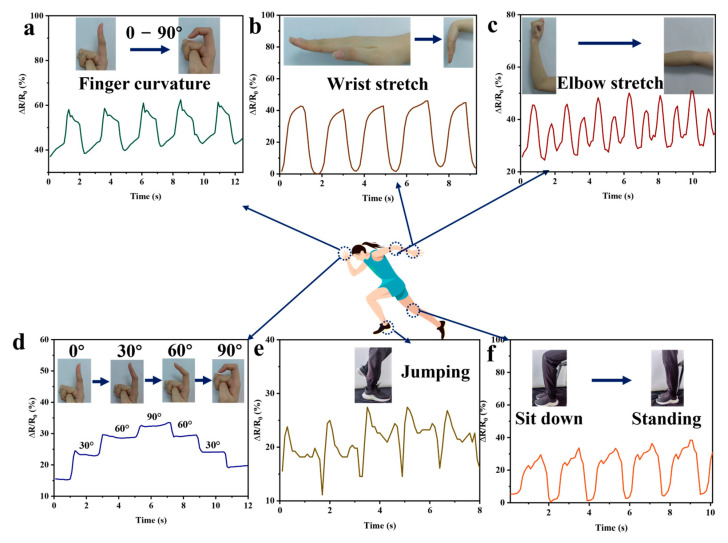
GG-CNF/SA/PAM is used to monitor different human activities in real-time. Finger 0–90° bending repetitions (**a**); wrist 0–90° repetitive stretching (**b**); elbow 0–90° stretch repetitions (**c**); fingers with different degrees of bending (**d**); jumping (**e**); and knee bending–stretching (**f**).

GG-CNF/SA/PAM is pasted on different body parts to detect the change in resistance (∆R/R_0_) with movement. As shown in Figure 9a,b, when the finger is at 0–90° and the wrist is stretched–compressed, different changes in resistance can be output to clearly identify whether it is a finger or wrist movement. The difference between inwardly tucked and outwardly extended elbows can also be clearly recognised in Figure 9c. Besides that, in Figure 9d, it is shown that the GG-CNF/SA/PAM can maintain the relative resistance change even during small movement changes. In Figure 9e, it was tested whether the hydrogel could respond quickly to transient stimuli, and the results showed that regular ∆R/R_0_ changes could still be obtained during repeated jumping–falling. Furthermore, when repeating the sitting down–standing up action, although it is a stretching–compressing action, the difference between the hydrogel affixed to the wrist, fingers, and knee can be clearly distinguished by the change in ∆R/R_0_ (Figure 9f). The stability of the hierarchical arrangement structure of the hydrogel network can provide faster electron/ion transport channels to accurately detect changes in resistance in response to different actions.

## 3. Conclusions

In conclusion, functional hydrogels (GG-CNF/SA/PAM) with hierarchical network structures were constructed by a one-step synthesis method. Graphene oxide (GO) modulated the hierarchical arrangement of the hydrogel using mechanisms such as the template effect and interfacial interactions, and nanofibrillated cellulose (CNF) entangled the overall network structure in a linear manner to enhance the stability of the hydrogel. In addition, the hierarchical hydrogel network structure still had good structural integrity after 100 compression cycles, and the cavities in the network structure were more likely to deform and return to their original shape when subjected to external forces. Meanwhile, it can provide faster electron/ion transport channels and enhance the mass transfer rate, so GG-CNF/SA/PAM has good sensing performance and changes in resistance with different actions. Furthermore, it is also expected to be used as a cold dressing material after sports strains. Compared with the traditional cold dressing, the temperature of the hydrogel can be adjusted by freezing temperature, and it can be plastically adhered to the skin surface at will. This feature of good resistance to drying, freezing, arbitrary plasticity, and accurate response to changes in resistance according to different actions provides a new way of thinking and a path for the research of flexible sensing and hydrogel sports dressings.

## 4. Materials and Methods

### 4.1. Materials

The nanofibrillated cellulose (CNF) used in this study was prepared in our previous studies [56,57] with a solid content of 8%. Sodium alginate (SA) was purchased from Shanghai Macklin Biochemical Co., Ltd. (Shanghai, China). Acrylamide (AM) was purchased from Fuchen Chemical Reagent Co., Ltd. (Tianjin, China). *N*,*N*′-methylenebisacrylamide (MBA) and ammonium persulfate (APS) were purchased from Shanghai Aladdin Biochemical Technology Co., Ltd. (Shanghai, China). Graphene oxide aqueous solution (GO, prepared by Hummers method, 2 mg/mL) was purchased from Suzhou Carbonfund Graphene Technology Co., Ltd. (Suzhou, China). Glycerol was purchased from Fuyu Fine Chemical Reagent Co., Ltd. (Tianjin, China). All chemicals used in this study were analytically pure and therefore did not require further purification. Deionised water was used throughout this study.

### 4.2. Preparation of Hydrogels

Initially, AM (2 g), APS (0.0168 g), and MBA (0.01 g) were added to 10 mL of CNF suspension and stirred for 2 h. To the above mixed solution, 0.05 g of sodium alginate (SA) was added and stirred until dissolved. Next, 5 mL of glycerol and 3 mL (2 mg/mL) of aqueous GO solution were added sequentially and stirring was continued for 6 h. The mixed solution obtained above was poured into a mould and sealed into a thermostat for 5 h (60 °C), named GG-CNF/SA/PAM. GO; GO and glycerol; and GO, glycerol, and CNF were removed sequentially, named G-CNF/SA/PAM, CNF/SA/PAM, and SA/PAM, respectively.

### 4.3. Characterisation of Hydrogels

Samples were freeze-dried using a freeze-dryer (Shuangjia Instrument Co., Ltd., Ningbo, China) for characterisation tests. The morphology and structure of the samples were characterised using a scanning electron microscope (SEM, Apreo S HiVac, Thermo Fisher Scientific, Waltham, MA, USA), a cryo-electron microscope (Cryo-EM, SU-8100, Hitachi Limited, Kyoto, Japan), and a transmission electron microscope (TEM, JEM-2100 JEOL Ltd., Kyoto, Japan), as well as to explore their micro-morphology and network structure. The samples were characterised using an X-ray diffractometer (XRD, 5–60°, 8°/min, XRD-6100, Shimadzu Corporation, Kyoto, Japan). The functional group profiles of the aqueous hydrogels were tested using a Fourier Transform Infrared Spectrometer (FTIR, samples scanned in the spectral range of 400–4000 cm^−1^, Spectrum 400, Perkin Elmer, Waltham, MA, USA). A differential scanning calorimeter (DSC Q20, Shanghai Leroy Scientific Instruments Co., Ltd., Shanghai, China) was used to test the sample state of the hydrogel at low temperature. Temperature was monitored using a thermal imaging camera (Testo 869, Testo, Schwarzwald, Germany). The dynamic modulus of the aqueous hydrogels was measured using a rotational rheometer (AR-2000ex, TA instruments, New Castle, DE, USA).

### 4.4. Anti-Drying Test

The hydrogels were placed in an oven at a constant temperature of 30 °C for 24 h and the weight of the hydrogels was monitored at fixed time intervals. In this way, the water retention properties of the hydrogels were examined [58,59]. Three groups were taken for each sample and the average value was taken at the completion of the test. The mass retention rate was calculated as follows:

(1) $$\mathrm{Quality}\;\mathrm{retention}\;\mathrm{rate}=\frac{W_{t}}{W_{0}}\times 100\%$$

where $W_{0}$ (g) is the initial weight of the hydrogel and $W_{t}$ (g) is the weight of the hydrogel at time t.

### 4.5. Swelling Test

The hydrogels dried for 24 h were immersed in deionised water and the weight of the hydrogels was monitored for a fixed period of time as a means of observing the network cross-linking state of the hydrogel re-swelling [60]. Three groups were taken for each sample, and the average value was taken at the end of the test. The swelling rate was calculated by the following formula:

(2) $$\mathrm{Swelling}\;\mathrm{rate}=\frac{W_{n}}{W_{0}}\times 100\%$$

where $W_{n}$ is the weight of the hydrogel at time n.

### 4.6. Mechanical Test

The mechanical properties of the hydrogels were tested using a universal mechanical testing machine (CMT 6104, Sansi Eternal Technology Co., Ltd., Ningbo, China). All the mechanical property experiments were carried out at 25 °C and 70% relative humidity. The dimensions of the hydrogels were fixed at 70 mm × 10 mm × 2 mm, and the tensile speed was 10 mm/min. In the compression experiments, the hydrogels were prepared in a cylindrical shape with fixed dimensions of 30 mm in height and 15 mm in diameter, and the compression speed was 5 mm/min. The experiments were repeated three times, and the average values were taken for further analysis.

### 4.7. Electrochemical Testing

The electrochemical performance testing method was used to test the sensing performance of the hydrogels using an electrochemical workstation (Chenhua CHI760e, Shanghai Chenhua Instrument Co., Ltd., Shanghai, China) (Test Methods in Ref. [30]). The dimensions of the hydrogel were prepared as 40 mm × 15 mm × 2 mm strips. The conductive copper wires were then fixed to the ends of the hydrogel, which was sandwiched in 3 M tape to prevent the evaporation of water. In addition, the above immobilised hydrogel was fixed in different positions of the body (fingers, elbows, wrists, soles, and knees). The electrode clips were then clamped onto the copper wires at each end of the hydrogel for current testing. The Init E (V) value was set to 0.1. During the test, the change in relative resistance (∆R/R_0_) was calculated and used to characterise the sensing performance of the hydrogel.

The conductivity of the hydrogel samples can be evaluated by the strain factor GF. The GF of the hydrogel is calculated by the following formula:

(3) $$\frac{∆R}{R_{0}}=\frac{R-R_{0}}{R_{0}}\times 100\%$$

(4) $$GF=\frac{\frac{∆R}{R}}{\varepsilon }\times 100\%$$

where $R_{0}$ (Ω) and R (Ω) are the resistance before and after applying the strain, respectively, ε is the value of strain change before and after stretching the hydrogel, and GF denotes the sensitivity factor.

## Data Availability

The original contributions presented in this study are included in the article. Further inquiries can be directed to the corresponding author.
